# Diagnosing Torsades De Pointes Based on Correlation to QT Interval: A Systematic Review

**DOI:** 10.7759/cureus.27833

**Published:** 2022-08-09

**Authors:** Simranjit S Kahlon, Rabia Sikandar, Sreedevi Tejovath, Shaalina Nair, Danial Hassan, Khushbu K Patel, Aishwarya Peddemul, Jihan A Mostafa

**Affiliations:** 1 Internal Medicine, California Institute of Behavioral Neurosciences & Psychology, Fairfield, USA; 2 Epidemiology and Public Health, Ministry of Public Health, Doha, QAT; 3 Cardiology, California Institute of Behavioral Neurosciences & Psychology, Fairfield, USA; 4 Obstetrics and Gynecology, California Institute of Behavioral Neurosciences & Psychology, Fairfield, USA

**Keywords:** qt prolongation and risk of torsades, drug-induced long qt syndrome, torsades, torsades de pointes (tdp), qt interval prolongation, long qt syndrome

## Abstract

Torsades de Pointes (TdP) is a rare form of tachyarrhythmia which can potentially be fatal due to its tendency to degenerate into ventricular fibrillation. It is described as a polymorphic ventricular tachycardia characterized by twisting of the QRS complexes around the electrocardiogram (ECG) baseline in patients with a prolonged QT interval. Prolonged QT interval is known as long QT syndrome.

Torsades de Poccurs most commonly in patients with an extended QT interval duration, and even though monitoring an ECG can assist in its prevention, there is no defined duration of a QT interval that can lead to an increased risk of Torsades de Pointes. So, it is hard to determine what QT interval constitutes enough risk for Torsades de Pointes to require intervention. The QT interval duration also depends on other factors, namely heart rate (HR) and other factors such as drugs, congenital diseases, and a combination of both.

In this study, we considered various causes of QT prolongation but mainly focused on congenital diseases, drugs, or perioperative risk of QT prolongation and the correlation with the risk of impending TdP. By following the Preferred Reporting Items for Systematic Reviews and Meta-Analyses (PRISMA) guidelines and researching studies on various databases, namely PubMed, Science Direct, Medline, and CiNii we were able to find various systematic reviews and articles showing the association between prolonged QT interval and its degeneration into TdP. This review encourages further research into this topic to understand the implications of QT prolongation and how it can help save the lives of patients with known long QT syndrome, or those on QT prolonging drugs with simple ECG monitoring and treatment for the respective cause.

## Introduction and background

*“The problem with heart disease is that the first symptom is often fatal.”* - Michael Phelps [1].

**Figure 1:**
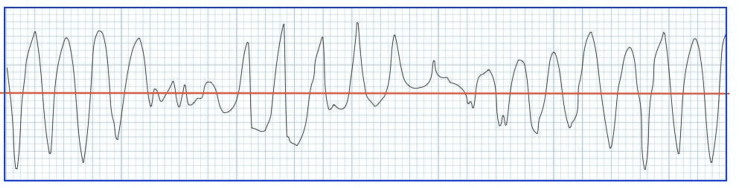
ECG showing Torsades de Pointes Image adapted with permission from Dr. Mike Cadogan, co-founder of Life in the Fast Lane (LITFL).

Torsades de Pointes (TdP) is described as a polymorphic ventricular tachycardia characterized by oscillatory changes in amplitude of the QRS complexes around the isoelectric line of the electrocardiogram (ECG) (Figure 1) and is preceded by an extended QT interval [2]. The corrected QT interval (QTc) is considered prolonged if > 440ms in men or > 460ms in women. Torsades de Pointes can potentially be fatal due to its tendency to degenerate into ventricular fibrillation. 

Despite QT prolongation being a risk factor related to an accelerated chance of TdP, there is no dependable criterion to discover the length of QT prolongation related to an increased risk of TdP. This makes it hard to determine what QT interval constitutes enough risk of TdP to require intervention. As QT interval by itself is partially dependent on heart rate (HR), the interpretation of QT intervals can be challenging. For instance, higher values of HR are related to a shorter QT interval and similarly, lower values of HR are naturally related to longer QT intervals. Hence, based totally on the dependence of QT on HR, a corrected QT interval (QTc) is estimated at an HR of 60 bpm. Correcting (Table 1), and interpreting, a cut-off value of QTc that defines the 'at-risk setting', is still debated [3].

**Table 1:** Formulas for corrected QT interval. The QT interval is specified in milliseconds and the RR interval in seconds. QTc: Corrected QT interval

Correction	Formula
Bazett’s correction	QTc = QT/RR0:5
Fridericia’s correction	QTc = QT/ RR0:33
Framingham's correction	QTc = QT + 0.156 x (1-RR)

**Figure 2:**
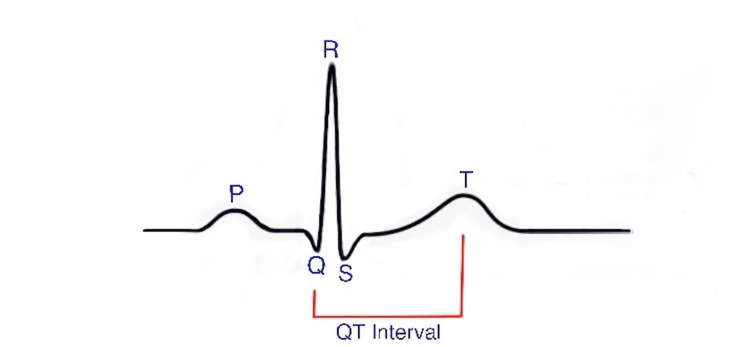
Normal ECG pattern with defining QT interval ECG: Electrocardiogram

Torsades de pointes most commonly arise in patients with a prolonged QT interval (Figure 2), also known as long QT syndrome, which is caused by irregular cardiac repolarization [3-5]. The long QT syndrome may be innate or acquired, or also a combination of both. The QT interval prolonging drugs usually leads to TdP. There is a sturdy correlation between a prolonged QTc and the clinical risk of developing TdP, however, there is no fixed threshold for QTc above which TdP automatically taSeveral drugs aregs that are routinely prescribed inside the perioperative setting, including antibiotics, antihistamines, antidepressants, antibiotics, anti-fungal drugs, antipsychotics, antiemetics, and general anesthetics that cause prolongation of the QTc interval. Moreover, in a recent study [6], it was discovered that 80% of patients undergoing non-cardiac surgical procedures under general anesthesia evolved postoperative QTc prolongation.

Medications that can cause QT prolongation, delay repolarization allowing early after-depolarizations, leading to a probable fatal ventricular arrhythmia called Torsades de pointes. Most medicinal drugs that extend the QT interval, act via binding to and inhibiting the potassium channel, thereby blockading the rapid component of the delayed rectifier potassium current (IKr) leading to a prolonged repolarisation phase of the cardiac action potential [7]. Repolarization is likewise maintained through other currents, and it is believed that people with impaired function of these additional currents (along with IKs) are at more hazard of drug-triggered QT prolongation and TdP [8]. This state, referred to as one of reduced repolarization reserve [7], may be brought on by risk factors that include congenital long QT syndrome, hypokalemia, and hypomagnesemia. Bradycardia, heart failure, and other risk factors (Table 2) also promote TdP. 

**Table 2:** Risk factors associated with TdP TdP: Torsades de Pointes, CYP3A4: Cytochrome P450 3A4, CHF: Congestive heart failure, MI: Myocardial infarction

Risk factors	Examples
Genetic	Channelopathy, CYP3A4 poor metabolizer
Secondary to cardiac diseases	Bradycardia, CHF, MI, Atrial fibrillation
Electrolyte derangement	Hypokalemia, Hypomagnesaemia, Hypocalcemia
Other systemic diseases	Renal Insufficiency, Severe hepatic disease
Medications	Antiarrhythmics, Antidepressants, Antipsychotics

Even though ECG monitoring can assist in preventing TdP. Adequate knowledge of ECG tracking in this setting is crucial for informed decision-making. A prolonged QT interval at a specific length can be used for the diagnosis of an impending TdP and thus provide a predictor that can save the lives of many patients with the risk of TdP. Therefore, we wrote a systematic review supporting the correlation between QT prolongation and the clinical risk of TdP [9].

## Review

Method

Search Strategy 

In this study, a search of scientific papers and statements known to the authors was conducted on PubMed, Science Direct, CiNii, and Medline with the following main keywords (Table 3): “QT prolongation”, “Torsades”, “Torsades de pointes”, “Long QT syndrome” and QT prolongation and risk of Torsades de pointes” was performed to determine the correlation between QT prolongation and the clinical risk of Torsades de Pointes. The MeSH keyword generated was (( "Long QT Syndrome/analysis"[Mesh] OR "Long QT Syndrome/anatomy and histology"[Mesh] OR "Long QT Syndrome/classification"[Mesh] OR "Long QT Syndrome/complications"[Mesh] OR "Long QT Syndrome/diagnosis"[Mesh] OR "Long QT Syndrome/diagnostic imaging"[Mesh] OR "Long QT Syndrome/drug therapy"[Mesh] OR "Long QT Syndrome/etiology"[Mesh] OR "Long QT Syndrome/history"[Mesh] OR "Long QT Syndrome/metabolism"[Mesh] OR "Long QT Syndrome/pathology"[Mesh] OR "Long QT Syndrome/physiology"[Mesh] OR "Long QT Syndrome/physiopathology"[Mesh] OR "Long QT Syndrome/prevention and control"[Mesh] OR "Long QT Syndrome/statistics and numerical data"[Mesh] )) AND ( "Torsades de Pointes/analysis"[Mesh] OR "Torsades de Pointes/chemically induced"[Mesh] OR "Torsades de Pointes/complications"[Mesh] OR "Torsades de Pointes/diagnosis"[Mesh] OR "Torsades de Pointes/diagnostic imaging"[Mesh] OR "Torsades de Pointes/drug therapy"[Mesh] OR "Torsades de Pointes/etiology"[Mesh] OR "Torsades de Pointes/history"[Mesh] OR "Torsades de Pointes/metabolism"[Mesh] OR "Torsades de Pointes/mortality"[Mesh] OR "Torsades de Pointes/pathology"[Mesh] OR "Torsades de Pointes/physiology"[Mesh] OR "Torsades de Pointes/physiopathology"[Mesh] OR "Torsades de Pointes/prevention and control"[Mesh] OR "Torsades de Pointes/statistics and numerical data"[Mesh]).

**Table 3:** Search strategy

Name of Database	Keywords	Filter Criteria	Search Result
PubMed	(( "Long QT Syndrome/analysis"[Mesh] OR "Long QT Syndrome/anatomy and histology"[Mesh] OR "Long QT Syndrome/classification"[Mesh] OR "Long QT Syndrome/complications"[Mesh] OR "Long QT Syndrome/diagnosis"[Mesh] OR "Long QT Syndrome/diagnostic imaging"[Mesh] OR "Long QT Syndrome/drug therapy"[Mesh] OR "Long QT Syndrome/etiology"[Mesh] OR "Long QT Syndrome/history"[Mesh] OR "Long QT Syndrome/metabolism"[Mesh] OR "Long QT Syndrome/pathology"[Mesh] OR "Long QT Syndrome/physiology"[Mesh] OR "Long QT Syndrome/physiopathology"[Mesh] OR "Long QT Syndrome/prevention and control"[Mesh] OR "Long QT Syndrome/statistics and numerical data"[Mesh] )) AND ( "Torsades de Pointes/analysis"[Mesh] OR "Torsades de Pointes/chemically induced"[Mesh] OR "Torsades de Pointes/complications"[Mesh] OR "Torsades de Pointes/diagnosis"[Mesh] OR "Torsades de Pointes/diagnostic imaging"[Mesh] OR "Torsades de Pointes/drug therapy"[Mesh] OR "Torsades de Pointes/etiology"[Mesh] OR "Torsades de Pointes/history"[Mesh] OR "Torsades de Pointes/metabolism"[Mesh] OR "Torsades de Pointes/mortality"[Mesh] OR "Torsades de Pointes/pathology"[Mesh] OR "Torsades de Pointes/physiology"[Mesh] OR "Torsades de Pointes/physiopathology"[Mesh] OR "Torsades de Pointes/prevention and control"[Mesh] OR "Torsades de Pointes/statistics and numerical data"[Mesh] )	Article Type: Clinical Trial, Meta-Analysis, Random Clinical Trial (RCT), Review, Systematic Review Publication Dates: 1997 to 2022	78
Science Direct	QT prolongation AND Torsades AND Long QT Syndrome AND QT prolongation and risk of Torsades de pointes	Article Type: Review Article, Research Article Subject area: Medicine and Dentistry Publication Date: 2017-2022	819
Medline	QT prolongation AND Torsades AND Long QT Syndrome AND QT prolongation and risk of Torsades de pointes	All results	10
CiNii	QT prolongation AND Torsades AND Long QT Syndrome AND QT prolongation and risk of Torsades de pointes	All results	118

Inclusion Criteria

Articles were qualified for inclusion when they reported episodes of TdP occurring in close proximity to a prolonged QT interval whether congenital, drug-induced, or a combination of both in human patients. No age group was excluded.

Exclusion Criteria

Exclusion criteria included studies that were case reports, encyclopedias, educational lectures, case series, and abstracts presented at conferences and articles that do not fit the inclusion criteria. Articles in languages other than English were also excluded.

Ascertainment of Risk of Bias in Individual Studies

Specific tools for risk of bias ascertainment were used on articles that met the inclusion criteria. Assessment of multiple systematic reviews 2 (AMSTAR 2) for systematic reviews; meta-analyses, scale for the assessment of narrative review articles 2 (SANRA 2) for narrative reviews; Cochrane collaboration risk of bias tool (CCRBT) for randomized clinical trials (RCTs); the Newcastle-Ottawa scale (NOS) for cohort studies and non-randomized clinical trials (RCTs); and a score of at least 70% for each assessment tool was used as the benchmark of acceptance for the study.

Selection of Studies

In our systematic review, Preferred Reporting Items for Systematic Reviews and Meta-Analyses (PRISMA) guidelines were followed. Cases were considered from English sources. The search and article selection was primarily conducted by two authors. Typically, cases were selected in a straightforward manner, and initial disagreements were settled through consensus. The studies that met the inclusion criteria underwent a second round of screening after a full-text review and quality assessment tools. Seven case studies were selected for this review.

Results

**Figure 3:**
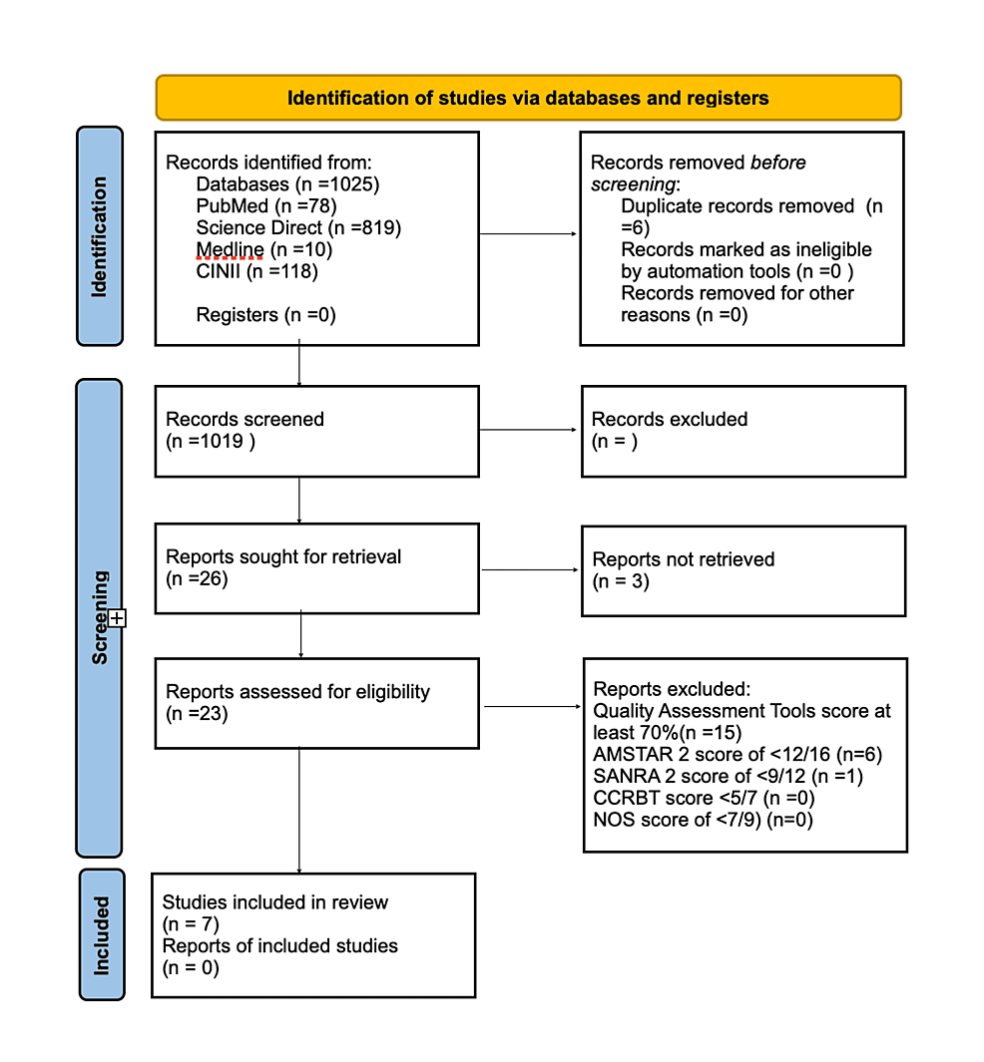
Identification of studies via databases and registers AMSTAR 2: Assessment of multiple systematic reviews 2, SANRA 2: Scale for the assessment of narrative review articles 2, CCRBT: Cochrane collaboration risk of bias tool, NOS: Newcastle-Ottawa scale

Articles were identified (Figure 3) through the literature review. The full texts of selected articles were obtained following initial screening. In particular, we excluded non-human studies, reviews, and comments that did not contain specific case data, insufficient evidence, and inadequate ECG measurement data.

In a study of QT prolongation and the risk of TdP [10], there was clear evidence of a pause-dependent TdP in 14 out of 15 patients (95% confidence interval (CI), 68% to 100%). The length of the cycle with pauses leading to TdP was 1.3+-2 times longer than the basic cycle length, and 80% of the pauses leading to TdP were longer than the basic cycle length (80 ms longer than the basic cycle length). The results suggest that this sequence, which has been recognized as a hallmark of TdP in the acquired long QT syndrome, plays a major role in the genesis of TdP in the congenital long QT syndrome as well [10]. Torsades de Pointes was consistently seen in almost all patients in the study group.

A basic RR interval of 830 _+67 ms was found in the 14 patients with pause-induced TdP (range 640 to 960) before any cycle length disturbance occurred, which is equivalent to a heart rate between 62 and 94 beats per minute. Premature beats (short cycle) initiating long-short sequences were coupling at 440 to 720Hz (p * 0.01 vs. cycle length). Finally, the mean RR interval of the pauses leading to increased ventricular ectopic beats culminating in TdP was 1,068 _+ 206 ms (range 760 to 1,560 ms, p < 0.01vs. basic cycle length). It is unambiguously evident that the vast majority of pauses leading to TdP have been exceedingly long: 96%, 90%, and 80% of pauses have been >40, >65, and >80 ms longer than the preceding basic cycle length, respectively. There was an average cycle length of 1.3 times +0.2 times longer for the pause (long cycle) leading to the TdP [10].

A study by Topilski et al. [11] showed there were no significant differences between the 30 patients with TdP and the 113 patients with uncomplicated bradyarrhythmias in terms of age, clinical background, and drug therapy, or potassium serum levels. The TdP risk was not predicted by either ventricular rate or QRS width at the time of worst bradyarrhythmia. However, a correlation was observed between the QT, corrected QT (QTc), and T-peak-T-end intervals, and the risk of TdP. A T-peak-T-end of 117 ms was found to be the best single discriminator. A morphology similar to LQT1 (long QT interval with broad T-waves) or LQT3 (small and late) was rarely seen in bradyarrhythmias. The study noted that LQT2-like notched T-waves were observed in 55% of patients with TdP but in only 3% of patients with uncomplicated bradyarrhythmias (p=0.001). Using a two-step model based on QT duration and the presence of LQT2-like T-waves, 84% of patients were predicted to develop TdP [11]. An extended QT interval, a prolonged QTc interval, and a prolonged T-peak-T-end interval are all associated with an increased risk of TdP during acquired bradyarrhythmias, especially when notched T-waves are present [11].

In a study of patients with perioperative TdP [6], 46 cases were identified, 29 of which were women (67%) and two of which were fatal (case fatality rate: 4%). Around 40% of the cases were related to craniotomies and cardiac surgery. According to the authors, hypokalemia and bradycardia were identified as preceding events and a third of the cases appeared to be caused by drugs. The mean QTc at baseline was 457 ± 67ms and at the time of the event, the mean QTc increased to 575 ± 77ms. An increase of +118ms (99% CI, 70 to 166 ms; p< 0.001) in the QTc was seen between baseline and after the TdP event. Except for two patients, all had a substantial prolongation of their QTc interval at the time of the event [6].

There was a correlation between the QT interval, QTc interval, and T-peak-T-end intervals and TdP risk. According to the receiver operating characteristic (ROC) curve analysis, T-peak-T-end intervals were the best single discriminator between patients with and without TdP. The T-peak-T-end area under the curve (which acts as a measure of sensitivity versus 1-specificity), the QTcs area under the curve, and the QT area under the curve were all 0.994. In addition, the positive predictive value (PPV) of QT and QTc intervals exhibited a linear trend, increasing as the QT interval (or QTc interval) increased. The -peak-T-end, on the other hand, exhibited an S-shaped curve, with a dramatic increase in risk as T-peak-T-end increased above 60 ms. There are two best cutoff values for determining whether a patient has TdP or a common bradyarrhythmia. In order to maintain 100% sensitivity while minimizing the risk of TdP, it may be prudent to compromise on specificity. In all TdP cases, QTc intervals between 400 ms and 510 ms were identified. This was also found to be the case in 36% and 49% of TdP patients, respectively.

Several characteristics were identified as associated with perioperative TdP in the systematic review and meta-analysis. Torsade de Pointes has been associated with the combination of several factors, such as hypokalemia, congenital long QT syndrome, and a drug that prolongs QT intervals. One episode of perioperative TdP was fatal (case fatality rate: 4%); 40% required magnesium treatment and one in four patients required defibrillation [6].

Discussion

The systematic review identified several studies associated with QT prolongation and the risk of Torsades de pointes. The association of several risk factors such as perioperative, hypokalemia, or congenital long QT syndrome and QT interval prolonging drug was identified as a trigger for the Torsades de pointes event. Many drugs are used in routine for different diseases that may QT prolongation in not only a susceptible person but also a healthy individual. A list of common drugs causing QT prolongation is shown in the following table (Table 4).

**Table 4:** Medications causing QT prolongation

Class	Drugs
Antiarrythmics	Disopyramide, procainamide, quinidine, sotalol
Antifungals	Ketoconazole, fluconazole, voriconazole, pentamidine
Antidepressants	Citalopram, escitalopram
Antipsychotics	Haloperidol, thioridazine
Fluoroquinolones	Ciprofloxacin, levofloxacin, moxifloxacin
Macrolides	Azithromycin, clarithromycin, erythromycin
Opioids	Methadone
Antiemetics	Granisetron, ondansetron
Others	Cocaine, cilostazol, donepzil

QT Prolongation and Torsades de Pointes: Relationship with Drugs

A systematic analysis of drug-induced TdP reported Torsades de Pointes in 14 of the 15 patients with QTc prolongation, which is 93.3% of the patients with a 95% confidence interval. The Bazette formula for QT correction has shown high sensitivity but the QT nomogram is highly sensitive and specific for Drug-induced TDP. Especially since the Bazette formula shows inaccuracies below 60 heartbeats per minute. The Bazette formula and QT nomogram are similar at QTc of 500ms and 70 to 100 heartbeats per minute. The results were very significant despite the small sample size of the study.

A number of synergistic mechanisms lead to long QT syndrome: (1) An increase in the duration and dispersion of the action potentials [12,13] as well as the formation of early afterdepolarizations are a couple of ways that adrenergic stimulation can enhance ventricular ectopic beating [12,14]; (2) As a result of preterm ventricular complexes, postextrasystolic pauses are generated, causing further delayed repolarization [15] and increased refractory period dispersion [16]; (3) Further triggering of triggered activity is enhanced by the RR intervals of post-extrasystolic pauses [17,18], and increased sympathetic tone facilitates the conduction of these early depolarizations from their origin to the surrounding myocardium to initiate TdP [10]. In some cases of excessive QT prolongation or extreme stress, adrenergic-mediated early afterdepolarizations could cause TdP directly. An initiation of TdP in the congenital form of long QT syndrome is usually impossible without a long-short sequence. There is still debate as to whether this polymorphic ventricular tachyarrhythmia results from multiple triggering waves or reentry [16,17,18].

The long-short sequence has been recognized as a hallmark of TdP in acquired long QT syndrome, but it also plays a role in bringing about TdP in those with congenital long QT syndrome [10]. The findings of the study have therapeutic implications regarding the use of pacemakers in the treatment of congenital long QT syndrome.

Congenital QT Prolongation and Risk of Torsades de Pointes

In another study of QT prolongation in patients with congenital QT Prolongation [14], the predictive value of TdP showed a positive predictive value of 84%. The TdP risk increases with prolonged QT intervals, QTc intervals, and T-peak-T-end during acquired bradyarrhythmias, particularly when accompanied by QT prolongation. The three types of T-waves were identified and classified as LQT1-like (long QT interval with broad T-waves), LQT2-like (notched T-waves), and LQT3-like (small and late) T-waves. During bradyarrhythmia, LQT2-like T-waves were observed in approximately 55% of the cases and only 3% in uncomplicated bradyarrhythmias (p 0.001) while LQT1 and LQT2 type T-waves were rare [14].

Currently, QT prolongation is not considered an indication for pacemaker implantation during acquired atrioventricular block or sinus node dysfunction by the American Heart Association/American College of Cardiology. The risk of developing TdP is higher for patients with prolonged QT intervals and QTc intervals. The rationalization of urgent pacemaker implantation even if symptoms or additional indications are absent is that the QT prolongation does not terminate immediately after the bradyarrhythmia is resolved. So, the recommendation is that a pacemaker is implanted and programmed until the QT interval shortens [14].

Perioperative Risk of QT Prolongation and Torsades de Pointes

Using the available evidence from 46 cases, we evaluated the perioperative risk of TdP and QT prolongation and found several pertinent findings. To start with, almost every episode of perioperative TdP occurs with a prolonged QTc interval. At the time of the event, the majority of patients experienced an increase in QTc >100ms, while only two patients did not experience this significant increase. So, it appears that perioperative TdP is frequently caused by QTc prolongation, but by itself is not enough to trigger the actual episodes. According to the evidence, perioperative TdP is triggered by the simultaneous occurrence of several factors, including hypokalemia, bradycardia, or drug-drug interactions while QTc is prolonged. There have been several drug-induced causes cited for perioperative TdP [6].

Several common risk factors for perioperative Torsades de pointes were identified in this systematic review. The nearly universal presence of a QTc interval prolongation following TdP suggests that perioperative QTc interval prolongation should be closely monitored.

There were some common characteristics among the 46 cases of perioperative TdP in this systematic review. It was common for several QT-prolonging factors to contribute to virtually all episodes of QTc prolongation. It may be prudent to pay more attention to the potential for QTc interval prolongation and TdP during perioperative surgery given the typical exposure to drugs and physiological stressors that may affect myocardial repolarization.

Pacemakers were used in bradyarrhythmias caused by various factors more specifically beta-blockers used in young adults with congenital long QT syndrome but recent studies suggest the benefits of using a permanent pacemaker for long-term pacing even in patients without bradyarrhythmias. Although the QTc interval still remains prolonged after pacing it has shown evidence of the benefits of long-term pacing. An alternative method to prevent pause-induced TdP is to use pacing algorithms specifically designed for its prevention. Several pacemakers are equipped with a feature that detects premature complexes.

Miscellaneous Causes of QT Prolongation and Torsades de Pointes

Many other systematic reviews and articles showed the co-relation of specific drugs and diseases including Covid-19 causing prolonged QT intervals and subsequent TdP often leading to fatal ventricular fibrillation. Many drugs mentioned above in Table 4 have been studied individually in the causation of QT prolongation. Hence, providing further evidence of a correlation between QT prolongation and the risk of TdP. 

Limitations

This study may have several limitations as the quality of this review is dependent on the available studies and published systematic reviews. There were no uniform reporting standards for perioperative TdP, so missing data were a substantial limitation. While our search for TdP cases was systematic, it was not extensive. We did not search databases of spontaneously reported adverse drug reactions, nor did we contact pharmaceutical companies for any additional information. An in-depth search, on the other hand, would likely lead to a stronger relationship and a more conclusive outcome.

## Conclusions

The study found evidence of a co-relation between QT prolongation and the clinical risk of Torsades de pointes whether it be congenital, drug-induced, or a combination of these. Multiple risk factors including perioperative, hypokalemia, or congenital long QT syndrome and QT interval-prolonging drugs of both have been implicated in the causation of TdP, and further life-threatening ventricular defibrillation which may often be fatal. Nearly all episodes were preceded by significant QTc prolongation due to multiple QTc-prolonging factors. As a result of the limited evidence in the current literature, prevention strategies such as QTc monitoring ought to be considered in all patients.
